# The expanding universe of transposon technologies for gene and cell engineering

**DOI:** 10.1186/1759-8753-1-25

**Published:** 2010-12-07

**Authors:** Zoltán Ivics, Zsuzsanna Izsvák

**Affiliations:** 1 Max Delbrück Center for Molecular Medicine, Berlin, Germany; 2 Department of Microbial Biotechnology and Cell Biology and Department of Human Genetics, University of Debrecen, Debrecen, Hungary

## Abstract

Transposable elements can be viewed as natural DNA transfer vehicles that, similar to integrating viruses, are capable of efficient genomic insertion. The mobility of class II transposable elements (DNA transposons) can be controlled by conditionally providing the transposase component of the transposition reaction. Thus, a DNA of interest (be it a fluorescent marker, a small hairpin (sh)RNA expression cassette, a mutagenic gene trap or a therapeutic gene construct) cloned between the inverted repeat sequences of a transposon-based vector can be used for stable genomic insertion in a regulated and highly efficient manner. This methodological paradigm opened up a number of avenues for genome manipulations in vertebrates, including transgenesis for the generation of transgenic cells in tissue culture, the production of germline transgenic animals for basic and applied research, forward genetic screens for functional gene annotation in model species, and therapy of genetic disorders in humans. *Sleeping Beauty *(*SB*) was the first transposon shown to be capable of gene transfer in vertebrate cells, and recent results confirm that *SB *supports a full spectrum of genetic engineering including transgenesis, insertional mutagenesis, and therapeutic somatic gene transfer both *ex vivo *and *in vivo*. The first clinical application of the *SB *system will help to validate both the safety and efficacy of this approach. In this review, we describe the major transposon systems currently available (with special emphasis on *SB*), discuss the various parameters and considerations pertinent to their experimental use, and highlight the state of the art in transposon technology in diverse genetic applications.

## Transposons as genetic tools

**Figure 1:**
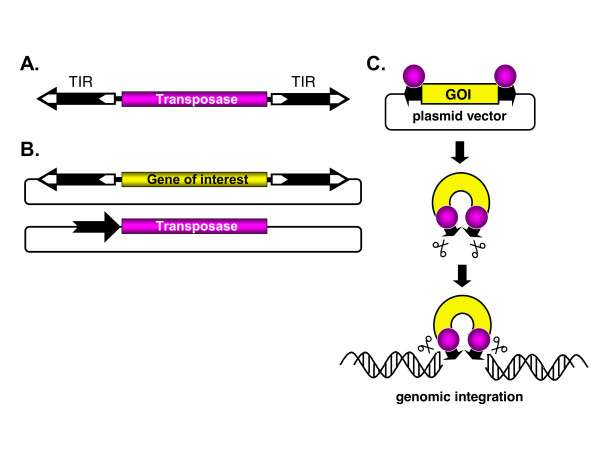
**General organization and use of class II transposable elements as gene vectors**. (**A**) Autonomous transposable elements consist of terminal inverted repeats (TIR; black arrows) that flank the transposase gene. (**B**) Bi-component transposon vector system for delivering transgenes that are maintained in plasmids. One component contains a DNA of interest between the transposon TIRs carried by a plasmid vector, whereas the other component is a transposase expression plasmid, in which the black arrow represents the promoter driving expression of the transposase. (**C**) The transposon carrying a DNA of interest is excised from the donor plasmid and is integrated at a chromosomal site by the transposase.

**Figure 2:**
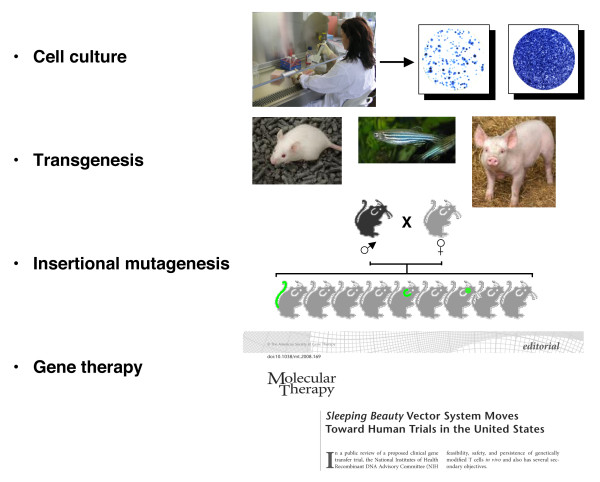
**Broad applicability of transposon-based gene vectors in vertebrate genetics**.

DNA transposons are discrete pieces of DNA with the ability to change their positions within the genome *via *a 'cut and paste' mechanism called transposition. In nature, these elements exist as single units containing the transposase gene flanked by terminal inverted repeats (TIRs) that carry transposase binding sites (Figure 1A). However, under laboratory conditions, it is possible to use transposons as bi-component systems, in which virtually any DNA sequence of interest can be placed between the transposon TIRs and mobilized by *trans*-supplementing the transposase in the form of an expression plasmid (Figure 1B) or mRNA synthesized *in vitro*. In the transposition process, the transposase enzyme mediates the excision of the element from its donor plasmid, followed by reintegration of the transposon into a chromosomal locus (Figure 1C). This feature makes transposons natural and easily controllable DNA delivery vehicles that can be used as tools for versatile applications, ranging from somatic and germline transgenesis to functional genomics and gene therapy (Figure 2).

Transposons have been successfully used in plants and in invertebrate animal models, including *Arabidopsis*, rice, *Caenorhabditis elegans *[1-3] and *Drosophila *[4-6] for transgenesis and insertional mutagenesis, but until recently, there was no known transposon that was sufficiently active to be tailored as a tool for such purposes in vertebrates. This is because transposons tend to have limitations with respect to the species in which they can jump. In 1997, the *Sleeping Beauty *(*SB*) transposon system was engineered by molecular reconstruction of an ancient, inactive Tc1/*mariner*-type transposon found in several fish genomes [7]. This newly reactivated element allowed highly efficient transposition-mediated gene transfer in major vertebrate model species without the potential risk of cross-mobilization of endogenous transposon copies in host genomes. This is because the genomes of major models lack endogenous transposon sequences with sufficient sequence similarity for mobilization by an exogenously supplied *SB *transposase. Indeed, *SB *has been successfully used as a tool for genetic modifications of a wide variety of vertebrate cell lines and species including humans [8-10].

During the past decade, other elements have been shown to catalyze efficient transposition in vertebrate model organisms. For example, the insect elements *piggyBac *[11,12] and *Minos *[13,14] catalyze efficient transposition in mammalian cells. *Minos *was also shown to be active in the basal chordate *Ciona intestinalis *[15]. Moreover, the reconstructed amphibian element *Frog Prince *[16], the reconstructed human *Hsmar1 *element [17], the reconstructed zebrafish transposon *Harbinger3_DR *[18], and the *Tol1 *[19] and *Tol2 *[20] elements isolated from the medaka fish have been found to be active in vertebrate species. *Passport*, a Tc1-family transposon isolated from a fish (*Pleuronectes platessa*), is active in a variety of vertebrate cells [21], and the *Ac/Ds *transposon originally discovered in maize by McClintock undergoes efficient transposition in zebrafish embryos [22]. Thus, the *piggyBac*, *Minos *and *Ac/Ds *elements appear to have a significantly wider possible host range than most other transposons. The basic criteria for the applicability of a transposon in any given model organism are 1) a sufficient level of transpositional activity in the given species, and 2) target site selection properties of the transposon, which are discussed below.

### Hyperactive transposon systems

In evolutionary terms, the *SB *transposon represents a successful element that was able to colonize several fish genomes millions of years ago [7]. However, even successful transposons have not been selected for the highest possible activity in nature, because unlike viruses, they have to coexist with their hosts and, consequently, there is strong selective pressure to avoid insertional mutagenesis of essential genes. Indeed, although the resurrected *SB *element was sufficiently active to be mobilized in vertebrate cells, its relatively limited transpositional activity still presented a bottleneck for some applications. For example, requirements for transfection of primary cells and other hard-to-transfect cell types, or for remobilization of transposons from chromosomally resident single-copy donor sites, demanded an enzyme with more robust activity. Thus, enhancing transpositional activity has been one of the main targets for transposon vector development. To date, almost every single amino acid in the *SB *transposase has been changed in an attempt to increase its activity. Three main strategies have been applied to derive hyperactive mutants of the *SB *transposase: 'importing' amino acids and small blocks of amino acids from related transposases [23-25], systematic alanine scanning [26], and rational replacement of selected amino acid residues [24]. Together, these studies have yielded several, single amino acid replacements, each resulting in a relatively modest increase in transpositional activities. Unfortunately, the hyperactivity of most of the *SB *transposase mutants selected in immortalized cell lines did not translate to efficient stable gene transfer in primary cells *in vivo *[25,26].

Earlier studies established that certain combinations of amino acid replacements, each leading to hyperactivity, can yield a further enhancement in transpositional activity of the *SB *transposase [23-26], but guessing the correct combinations of variants out of the millions that are possible is like finding the correct combinations of numbers in a lottery. A high-throughput, PCR-based, DNA-shuffling strategy and screening of 2000 gene variants in mammalian cells produced a variant of *SB *that was 100-fold more potent in chromosomal insertion of a transgene than the originally reconstructed protein [27]. The use of *SB100X *demonstrated that it is possible to establish a transposon-based, non-viral vector system that is capable of stable gene transfer coupled with long-term gene expression at an efficiency comparable with that of viral strategies [27]. Thus, the hyperactive *SB100X *transposase holds great promise of offering broad utility in gene therapy and functional genomics.

### Integration site preference

The insertion pattern of most transposons is nonrandom, showing characteristic preferences for insertion sites at the primary DNA sequence level, and 'hotspots' and 'cold regions' on a genome-wide scale. For example, for the primary DNA sequence, the *Tol2 *element does not appear to exhibit a pronounced preference for any sequence for insertion [28]. By contrast, the *Harbinger3_DR *transposon is highly specialized to integrate into the palindromic AAACACCWGGTCTTT consensus sequence [18], the *piggyBac *transposon targets the sequence TTAA, and all Tc1/*mariner *transposons, including *SB*, *Frog Prince*, *Minos *and *Hsmar1*, target their integration into TA dinucleotides. In the case of *SB*, this preference has been studied in detail, and palindromic AT repeats found to be the preferred sites for integration [29]. However, computational analyses revealed that target selection is determined primarily at the DNA structure level, not by specific base-pair interactions. For example, protein-induced deformability was shown to be associated with preferred *SB *insertion sites, whereas *piggyBac *and *Tol2 *integration sites lack such consistent, clear-cut structural patterns [30,31]. This suggested that integrations of *SB *will occur into any DNA available, depending on these preferences only, but this is not the case. In the context of chromatin, Tc1/*mariner *elements have no or weak preference for transcription units, the 5' regulatory regions are not favored, and most hits in genes are localized within introns [29,32]. By contrast, *piggyBac *shows a greater propensity to integrate into transcription units, with a preference for insertion around transcription start sites [12,33-35], and the *Tol2 *transposon also shows a pronounced preference for integration close to transcriptional start sites [28]. This control of integration at the chromatin level is poorly understood. One possible explanation for this is the interaction of the transposase with unknown, chromatin-associated factors. Supporting this hypothesis, it has been shown that a host-encoded protein, lens epithelium derived growth factor (LEDGF), is involved in directing integration of human immunodeficiency virus (HIV) into active genes [36]. Taken together, the preferences of particular elements to integrate into expressed genes versus non-coding DNA, and their preferences for integration sites within genes are expected to be substantially different.

Integration site preference can greatly influence the utility of transposon vectors for different applications. For example, human gene therapy protocols require application of transposon vectors showing the least preference for target genes, for obvious safety reasons. The *SB *system (which shows close-to-random insertion site distribution) appears to best satisfy these needs, whereas the *piggyBac *and *Tol2 *systems (which prefer genes and their upstream regulatory regions for insertion) appear to be less favorable for potential therapeutic applications. Nevertheless, a systematic assessment of potential genotoxic effects associated with genomic integration of transposon vectors will need to be performed either in cell-based assays and/or in animal models to provide clinically relevant data.

Unlike in therapeutic applications, hitting genes by insertional elements is the goal with forward mutagenesis screens. However, the insertional biases associated with vector systems represent the main limitation to full genome coverage with individual transposon-based vectors. Thus, in this respect, the utility of transposons for mutagenesis is greatly enhanced by the availability of multiple alternative vector systems with distinct preferences for insertion, such as *SB*, *Tol2 *and *piggyBac*. Indeed, the propensity of *Tol2 *to insert close to transcriptional start sites of genes might be particularly advantageous for enhancer trapping [37,38], while the propensity of *piggyBac *to insert into transcription units supports genome-wide mutagenesis with gene trap cassettes [39].

### Local hopping

'Local hopping' describes a phenomenon of chromosomal transposition in which transposons have a preference for landing into *cis*-linked sites in the vicinity of the donor locus. Local hopping seems to be a shared feature of 'cut and paste' transposons. However, the actual extent of hopping to linked chromosomal sites and the interval of local transposition varies. For example, the *P*-element transposon of *Drosophila *prefers to insert within ~100 kb of the donor site at a rate ~50-fold higher than in regions outside that interval [40]. Similarly, in germline mutagenesis screens in mice using *SB*, 30-80% of the transposons re-insert locally on either side of the transposon donor locus [41-43]. In contrast to the *P*-element, *SB *seems to have a much larger local transposition interval between 5 and 15 Mb [42].

The local hopping feature not only differs between different transposons, but a given transposon may show great variations in local hops in different hosts, and in different donor loci even in the same host. For example, about 50-60% of the reinserted *Ac *elements were found to be distributed within a 5-cM distance of the donor site in maize [44,45], and the frequency of local hopping greatly varies in *Arabidopsis *and tobacco, depending on the chromosomal location of the donor site [46-48]. Moreover, local hopping of the *Ac *element in tomato seems overall to be less prevalent than in maize [49,50], and there are species-specific differences in its tendency for local hopping out of different transposon donor loci [51]. This variation in local hopping of the same element could possibly be explained by varying affinity of the transposase to unknown, chromatin-associated factors in different hosts [52].

Local hopping can play a significant role in mutagenesis using chromosomally resident transposons. In practical terms, local hopping limits the chromosomal regions accessible to a transposon jumping out of a given chromosomal site [53]. To circumvent this limitation, establishing numerous 'launch pads' to initiate transposition out of different loci can be a viable strategy to increase coverage of gene mutations. On the other hand, local hopping can be useful for saturation mutagenesis within limited chromosomal regions.

## Transposons and functional genomics

**Figure 3:**
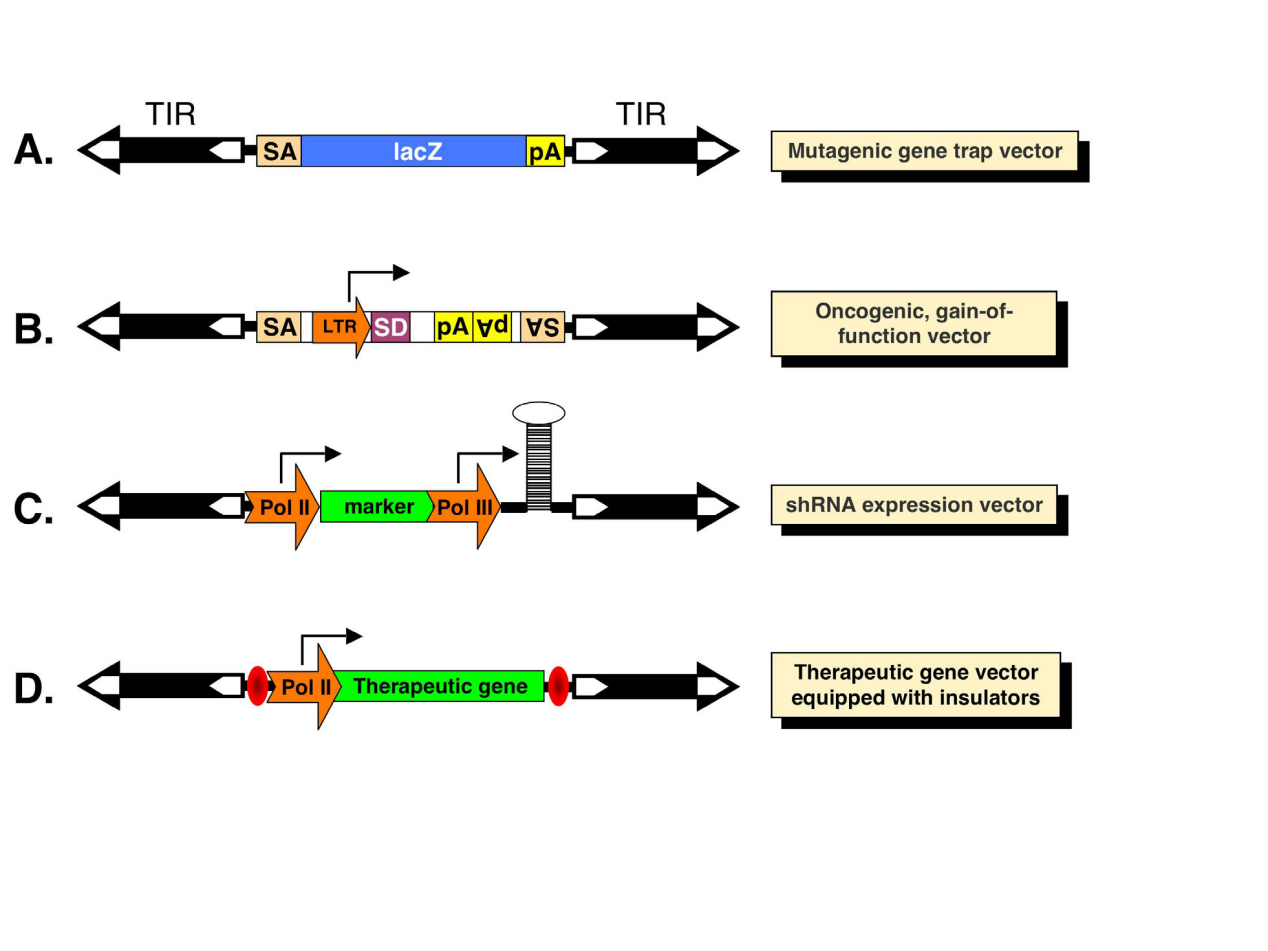
**Major types of expression and mutagenic cassettes delivered by transposon vectors for versatile applications**. (**A**) A gene-trap transposon contains a splice acceptor (SA) sequence followed by a promoterless reporter gene such as *lacZ *and a poly-A (pA) signal. The reporter gene is expressed only when its transcription is initiated from the promoter of a disrupted, actively transcribed endogenous transcription unit. Therefore, the expression pattern of the transposon reporter reflects that of the endogenous gene harboring the transposon insertion. (**B**) Oncogene-trap transposons include a strong viral promoter/enhancer long terminal repeat (LTR) sequence that can drive transcription toward the outside of the transposon and a splice donor (SD) sequence, so that forced transcription and splicing to downstream exons will result in overexpression of a product of a gene into which the transposon has inserted. Classic oncogene trap vectors also contain SA and pA sites to induce gene truncations that have dominant phenotypes. (**C**) Knockdown expression cassette including a polymerase (Pol) II promoter that drives expression of a marker gene and a Pol III promoter that drives expression of a short hairpin (sh)RNA. (**D**) A typical therapeutic expression cassette contains a ubiquitous or tissue-specific enhancer/Pol II promoter that drives expression of a therapeutic gene. To enhance the safety of such a vector, the expression cassette might be flanked by insulator elements that will block transactivation of endogenous promoters by the transposon insertion, and simultaneously protect the expression of the therapeutic gene from position effects.

The post-genomic era presented the scientific community with the new challenge of functional annotation of every gene and identification of elaborate genetic networks. Diverse methods have been employed to address this task, including mutational analysis, which proved to be one of the most direct ways to decipher gene functions. There are versatile strategies for creating mutations, including insertional mutagenesis by discrete pieces of foreign DNA, which has the advantage that the inserted DNA fragment can serve as a molecular tag that allows rapid, usually PCR-based, identification of the mutated allele. Because the function of the gene in which the insertion has occurred is often disturbed, such loss-of-function insertional mutagenesis is frequently followed by functional analysis of mutant phenotypes. In many instances, retroviral vectors were used to introduce mutagenic cassettes into genomes, but their chromosomal insertion bias does not allow full coverage of genes [54]. The random integration pattern of the *SB *transposon, combined with its ability to efficiently integrate versatile transgene cassettes into chromosomes established this system as an extremely useful tool for insertional mutagenesis in both embryonic stem cells (ESCs) [34,55] and in somatic [56,57] and germline tissues [41,42,53,58-63] in animal models (Figure 2). There are several types of mutagenic cassettes that can be efficiently combined with transposon-based gene delivery for insertional mutagenesis. 5' gene-trap cassettes include splice acceptors and polyadenylation sequences so that transcription of genes can be disrupted upon vector insertion into introns (Figure 3A) [54]. Often, such cassettes are also equipped with a reporter gene (usually, a fluorescent protein, β-galactosidase or antibiotic resistance) whose expression is dependent on correct splicing between exons of the trapped gene and the splice acceptor site carried by the transposon vector [64,65].

Insertional mutagenesis can be applied to cultured, germline-competent stem cells including ESCs and spermatogonial stem cells (SSCs) [39,66]. One advantage of this approach is the ability to perform preselection of modified ESC clones before generating mutant animals, and to differentiate selected clones into many different tissue types *in vitro*. It is possible to perform large-scale, transposon-based, insertional mutagenesis screens in ESCs and SSCs by simply transfecting or electroporating transposon donor and transposase expression plasmids into the cells. The amounts of the delivered plasmids can be adjusted to obtain the desired insertion frequencies per cell. In addition, transposons can also be remobilized from chromosomally resident loci and reintegrated somewhere else in the genome by transiently providing the transposase source; such excision-re-integration events can be monitored using double selection systems, in which excision activates the first and re-integration activates the second selection marker [43].

**Figure 4:**
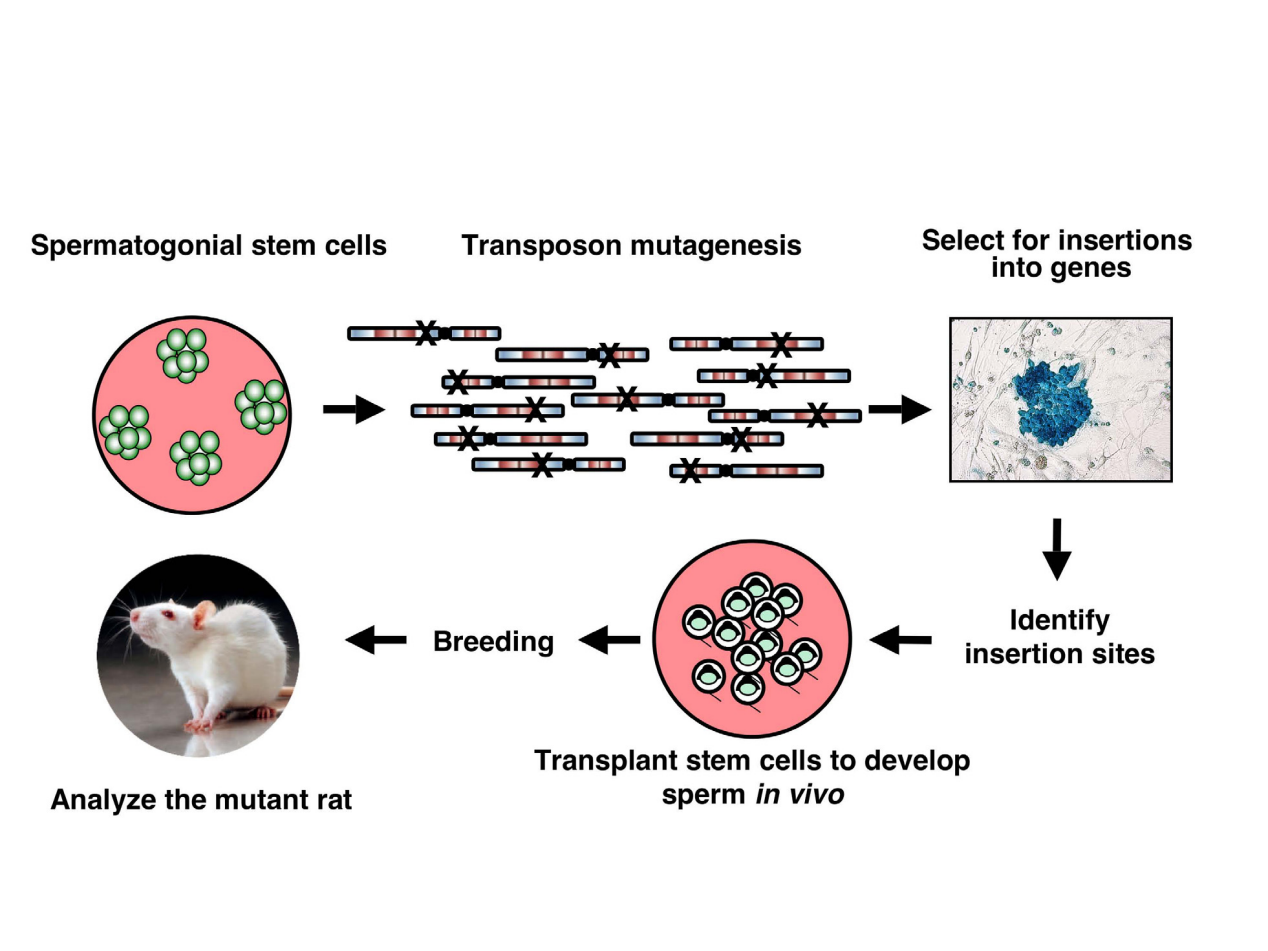
**Generation of knockout rats by insertional mutagenesis with gene trap transposons in spermatogonial stem cells**. Cultured stem cells are transfected with gene trap transposon and transposase constructs that will lead to thousands of transposon insertions covering all chromosomes. Those cells in which insertions have occurred in expressed genes can be selected based on activation of the gene trap marker, and the insertion sites can be mapped. Cell clones or polyclonal insertion libraries can be transplanted into the testes of sterile males, in which the spermatogonial step cells will undergo spermatogenesis. These transplanted males are crossed with wild-type females to pass the insertions through the germline and generate transgenic/knockout animals.

Because several aspects of physiology in rats have evolved to be more similar to humans than to mice, it would be desirable to use rat models in the process of functionally annotating the human genome by identifying the causative relationships between genes and disease phenotypes. As an important step towards this goal, an approach of establishing *SB *transposon-mediated insertional mutagenesis in rat SSCs was recently reported [66]. *SB *transposition can be used to tag and simultaneously mutate thousands of genes in culture, using gene-trap cassettes. Importantly, culture conditions maintain the potential of genetically manipulated SSCs to produce viable sperm cells. In that study, spermatogonial clones were transplanted to repopulate the testes of sterilized, wild-type recipient male rats. The stem cell genome was then passed on to transgenic offspring upon crossing of the recipient males with wild-type females (Figure 4). Although transposition events in a given target gene occur by chance, the tissue culture conditions allow screening for a large number of events. Transposition-mediated gene insertion and cell culture conditions thus allow generation of libraries of gene knockouts in rat SSCs (Figure 4). This technology has the potential to develop powerful genomic tools for use in the rat, offering the opportunity to create a bridge between physiology and genomics.

Another method in which transposons are used for insertional mutagenesis in animal models employs a 'jump-starter and mutator' scheme [42,58,61]. In this arrangement, mutator transgenic lines carry *SB *transposon-based gene-trapping vectors in the form of multicopy concatemers, whereas a jump-starter line expresses the transposase preferentially in the male germline [41,64]. Crossing of the two lines results in transposition in the germline of the F1 double-transgenic males, which are then repeatedly crossed with wild-type females to segregate the transposition events that occurred in their sperm cells to separate F2 animals. In the mouse system, a single sperm cell of an F1 male contains, on average, two transposon insertions [58], and up to 90% of the F2 progeny can carry transposon insertions [61]. The applicability of this approach has been demonstrated by the identification of mouse genes with either ubiquitous or tissue-specific expression patterns [42,64,67,68]. Recently, a similar system for *SB *insertional mutagenesis was also established in rats [62,63].

One cautionary note of launching transposition out of transposon arrays is that recombination between newly transposed transposon copies and the donor concatemer could lead to unwanted genomic rearrangements, as observed by Geurts *et al*. [68]. The most likely explanation for the rearrangements is that transposition out of a concatemer generates new transposase binding sites, linked either in *cis *(in the case of local hops) or in *trans *(in the case of transposition onto other chromosomes). However, because some transposon copies remain at the original donor locus, transposase can recombine chromosomal sequences that are located between the individual transposon units by hybrid element transposition (that is, the end of one transposon pairs with the opposite end of another transposon at a different location) [69], leading to deletions and translocations. Such chromosomal rearrangements are unlikely to occur if a single-copy donor is used. Thus, transposon systems sufficiently active for efficient transposition out of single-copy donors might eliminate the need for concatemeric donor sites in animal breeding schemes. Indeed, the *Tol2 *element was demonstrated to show transposition at reasonable efficiencies when launched from singly-copy donor sites in transgenic zebrafish [70]. In this context, the newly developed *SB100X *hyperactive system might also prove useful in future genetic screens.

Forward genetic screens do not necessarily need to depend on the breeding scheme described above; in some cases, a reasonable throughput in generation of transposon insertion mutants can be achieved by introducing the mutagenic transposon into individual animals, such as in zebrafish. A gene trap mutagenesis screen was recently employed to uncover genetic determinants of nicotine response in zebrafish, through a behavioral genetic screening paradigm [71]. Using standard transposase-mediated transgenesis protocols, *Tol2*-based mutagenic vectors were co-injected into early zebrafish embryos by to generate a pool of mosaic F0 founder animals, which then underwent two successive rounds of crossing to generate homozygous mutant animals. Segregation of mutant animals from wild-type siblings was carried out using fluorescent reporters built into the gene trap cassettes. After profiling nicotine response in mutant versus wild-type fish, two mutants were identified out of a total of 102 fish lines screened [71]. This study emphasizes the utility of transposons for the discovery and functional annotation of genes relevant to human health in forward, phenotype-driven genetic screens in model species.

### Transposon-based screens for cancer gene discovery

**Figure 5:**
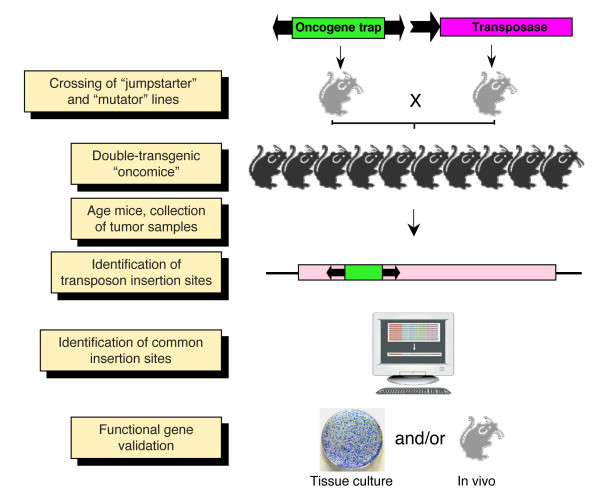
**Transposon-mediated cancer gene discovery screen**. Breeding of 'jumpstarter' and 'mutator' stocks induces transposition in the soma of double-transgenic animals ('oncomice'). In the case of tissue-specific screens, a third genotype containing a tissue-specific Cre allele has to be crossed in to the backgrounds. The crosses can be made either in wild-type or in specific cancer-predisposed genetic backgrounds. Transposition in somatic cells leads to random insertional mutations, and animals are monitored for tumor development. Transposon insertions are cloned from genomic DNA isolated from tumor samples, and are subsequently mapped and the mutagenized genes identified. Those genes repeatedly mutated in multiple, independent tumors are designated as common insertion sites (CIS). These candidate cancer genes are then functionally validated.

To induce gain-of-function mutations, transposon vectors can be equipped with oncogene trap cassettes that contain strong viral enhancers/promoters that can drive transcription outwards from the vector, thereby leading to overexpression of a full-length or truncated protein product of the trapped gene, as well as splice acceptor and polyA sites that lead to gene truncation with dominant phenotypes (Figure 3B) [9,72]. *SB *vectors harboring oncogene traps have been successfully used in large-scale cancer gene discovery screens in experimental animals (Figure 5) [10,73,74]. In these studies, *SB *transposons were somatically mobilized from donor chromosomal concatemers, which contained either low (25) [56] or high (150-350) [57] numbers of the oncogene trap transposon. Dominant mutations in somatic tissues of double transgenic mice carrying a transposase source and the mutagenic transposons resulted in the generation of experimental tumors in cancer-predisposed [56] and wild-type [57] animals. In a follow-up study, Collier *et al*. demonstrated that a combination of low-copy oncogene trap lines with the *SB11 *transposase (an early-generation hyperactive *SB *variant) expressed from the *Rosa26 *locus could achieve whole-body transposon mobilization at rates sufficient to promote penetrant tumorogenesis without complications of embryonic lethality or genomic instability [75]. Thus, this approach can be successfully employed not only to identify novel cancer genes, but also combinations of cancer genes that act together to transform a cell.

Current efforts are concentrating on customized, tissue-specific screens for cancer development studies. The strategies employed to achieve this goal focus on establishing mouse lines that either conditionally express the transposase from tissue-specific promoters, or rely on generation of Cre recombinase-inducible transposase alleles that can be used in conjunction with mice that express Cre in a tissue-specific manner [76-78]. For example, this approach was addressed by Dupuy and co-workers [72], who were able to experimentally modify the spectrum of tumors by creating a Cre-inducible *SB *transposase allele (RosaSBase^LsL^). With this strategy, they managed to overcome the obstacle of high embryonic lethality associated with ubiquitous *SB *transposase expression in the presence of the pT2/Onc2 oncogene trap [76,79], and to generate a model of germinal center B-cell lymphoma. They achieved this by activating *SB *transposase expression with an AidCre allele that drove Cre-mediated recombination in germinal center B-cells. In another approach, ubiquituous expression of the *SB *transposase was combined with the novel T2/Onc3 oncogene trap transposon vector. In that study, the MSCV (mouse stem cell virus) 5' long terminal repeat that was previously used to drive oncogene expression was replaced by the ubiquitously active CAGGS promoter, resulting in removal of the bias towards inducing mostly lymphomas and in reducing embryonic lethality. This strategy emphasizes that the change in the design of the mutagenic transposon (e.g. promoter choice) can have profound effects on the tumor types induced by transposition. Notably, this approach resulted in production of nearly 200 independent tumors of more than 20 types, and identification of novel, candidate cancer genes, suggesting that the combination of tissue-specific promoters and inducible transposase alleles could provide a fine mechanism of control in tumorogenesis studies.

## Transposons as vectors for stable transgene integration and expression

The classic approaches to stable expression of foreign genes in vertebrates rely on physical methods of delivering gene constructs into cultured cells, such as transfection, electroporation and sonoporation, or microinjection into oocytes or fertilized eggs to generate germline transgenic animals. The main drawbacks of these approaches are the low rates of genomic integration and the unstable expression of the chromosomally integrated gene construct, which is believed to be associated with the phenomenon of concatemerization of the injected DNA before genomic integration [80]. Another particular problem in transgenic animals is that founders that develop from the injected oocytes or eggs are predominantly mosaic for the transgene, because integration generally occurs relatively late during embryonic development. In principle, all of these drawbacks can be circumvented by transposition-mediated gene delivery, as it can increase the efficiency of chromosomal integration and facilitates single-copy insertion events. Single units of expression cassettes are presumably less prone to transgene silencing than are the concatemeric insertions created by classic methods.

Transposon-based technologies can be exploited for gene transfer in cultured cells and in primary cell types, including stem cells (Figure 2). For example, transposons can be harnessed to integrate plasmid-based shRNA expression cassettes into chromosomes to obtain stable knockdown cell lines by RNA interference (Figure 3C) [81]. Such technologies have been evaluated as a potential approach to the therapy of acquired immunodeficiency syndrome by stable RNA interference with *SB *vectors knocking down the CCR5 and CXCR4 cell surface co-receptors that are required for viral entry as a first step to confer resistance to HIV [82]. Both the *SB *and the *piggyBac *systems were shown to support efficient transposition in mouse [33,43] and human [83,84] ESCs. In a recent, elegant study, *piggyBac*-derived transgene vectors were introduced into human ESCs for the purpose of driving ESC differentiation toward a specific cell type [84]. The vectors included loss-of-function shRNA expression cassettes that could simultaneously knock down the expression of pluripotency genes and of genes that contribute to endodermal and mesodermal differentiation, plus a gain-of-function construct expressing Sox1 to direct differentiation towards neuroectoderm. What makes such complex gene-transfer experiments possible is the observation that several transgene constructs maintained on separate transposon vectors can be delivered simultaneously in 'multiplex' transposition reactions, in which the different constructs are simply mixed together and cotransfected into cells [85].

The recent discovery of induced pluripotent stem cells (iPSCs) by the expression of four key genes (*Oct4*, *Sox2*, *Klf4 *and c-*Myc*) in differentiated somatic cells holds enormous promise for future regenerative medicine [86]. Transposons are attractive vehicles for reversible production of iPSCs, because the excision step of the transposition reaction produced by transient re-expression of the transposase offers removal of the transgenes after completion of reprogramming, allowing subsequent differentiation of the iPSCs into various lineages *in vitro *[87]. Transposition-mediated generation of mouse and human iPSCs, and removal of the reprogramming factors from the pluripotent cells have already been achieved by the *piggyBac *system [88]. What makes *piggyBac *transposons especially attractive vectors for the production of transgene-free iPSCs is a special feature of these transposons: excision fully restores the sequence of the original wild-type locus [89], thereby allowing traceless removal of transgenes from the genome.

*In vivo*, co-injection of engineered transposons with transposase mRNA into fertilized oocytes can facilitate early integration events that potentiate successful transmission of the transgene through the germline to the next generation (Figure 2). This method has been employed to generate transgenic zebrafish with *Tc3 *[90], *Mos1 *[91], *Tol2 *[20] and *SB *[92]; transgenic *Xenopus *with *SB *[93] and *Tol2 *[94]; and transgenic mice with *SB *[27,95-97], *piggyBac *[11] and *Tol2 *[98]. In this context, an important step towards transgenesis with bacterial artificial chromosomes (BACs) has been made by the delivery of a ~70-kb BAC construct into zebrafish and mouse embryos with *Tol2 *[99]. Thus, transposons can evidently be used to stably deliver large transgene constructs together with complex regulatory regions, without the complications of DNA rearrangements and silencing associated with classic methods.

Any transgene vector system should provide long-term expression of transgenes. Transgenes delivered by non-viral approaches often form long, repeated arrays (concatemers) that are targets for transcriptional silencing by heterochromatin formation. In addition, long-term expression of transgenes delivered by retroviruses has been shown to be compromised by transcriptional silencing [100]. It was recently shown that the zinc finger protein ZFP809 bridges the integrated proviral DNA of the murine leukaemia virus and the tripartite motif-containing 28 transcriptional co-repressor in embryonic stem cells [101]. Thus, sequence elements in the vector itself can predispose the cargo for silencing. The cut and paste mechanism of DNA transposition results in a single copy of the transgene per insertion locus, thus concatemer-induced gene silencing is unlikely to be an issue with transposition-mediated gene transfer. Indeed, Grabundzija *et al*. found that transposon insertions delivered by the *SB*, *Tol2 *and *piggyBac *systems only rarely (< 4% of all insertions) undergo silencing in HeLa cells [28]. Furthermore, stable transgene expression observed in hundreds of independent insertions in this study suggests that these three transposon systems rarely target heterochromatic chromosomal regions for insertion, and that it is unlikely that certain sequence motifs in the transposon vectors are recognized by mediators of silencing in the cell. An additional factor that may provoke transgene silencing is the cargo DNA, particularly the type of promoter used to drive expression of the gene of interest. Indeed, it was previously shown that transgene constructs delivered into mouse cells using *SB *transposition can be subject to epigenetic regulation by CpG methylation and that a determinant of epigenetic modifications of the integrating transposon vector is the cargo transgene construct, with the promoter playing a major role [102]. However, with careful promoter choice, several studies have established that *SB*-mediated transposition provides long-term expression *in vivo*. For example, stable transgene expression from *SB *vectors was seen in mice after gene delivery in the liver [103-106], lung [107,108], brain [109] and blood after hematopoietic reconstitution *in vivo *[27,110]. Thus, although our understanding of all the factors that will ultimately determine the expressional fate of an integrated transposon is still rudimentary, it appears that transposon vectors have the capacity to provide long-term expression of transgenes both *in vitro *and *in vivo*.

## Transposons as vectors for gene therapy

Considerable effort has been devoted to the development of gene delivery strategies for the treatment of inherited and acquired disorders in humans. A desirable gene therapy approach should 1) achieve delivery of therapeutic genes at high efficiency specifically into the relevant cells, 2) be adaptable to changing needs in terms of vector design, 3) minimize the risk of genotoxicity, and 4) be cost-effective.

Adapting viruses for gene transfer is a popular approach; for example, γ-retroviral and lentiviral vectors are efficient at integrating foreign DNA into the chromosomes of transduced cells and have enormous potential for lifelong gene expression [111]. A major concern of using retroviral vectors is the potential for mutagenic effects at the sites of genomic integration [112-114]. Indeed, insertional mutagenesis has been observed in clinical trials using a retroviral vector for gene therapy of X-linked severe combined immunodeficiency [112,114,115]. The clinical use of retroviral vectors can be curtailed because of the limited size of the payload, as multiple or large transgenes compromise the efficiency of viral reverse transcription and packaging. Finally, regulatory issues and the high costs associated with manufacture of clinical-grade retrovirus hamper their widespread translation into clinical practice. An ideal therapeutic vector would combine the favorable attributes of integrating viral vectors (that is, stable chromosomal insertion) while significantly reducing the potential for adverse events. Transposons could potentially offer such an alternative (Figure 2).

The advantage of *SB *transposon-based gene delivery is that, owing to stable genomic insertion of expression cassettes, it can lead to both long-term and efficient transgene expression in preclinical animal models [116]. Thus, the *SB *plasmid-based transposon system combines the advantages of viral vectors with those of naked DNA molecules. However, in contrast to viral vectors, transposon vectors can be maintained and propagated as plasmid DNA, which makes them simple and inexpensive to manufacture, an important issue for the implementation of future clinical trials. Further advantages of the *SB *system include its reduced immunogenicity [103], no strict limitation of the size of expression cassettes [24] and improved safety and toxicity profiles [87,117-119]. Because the transposition mechanism does not involve reverse transcription, DNA-based transposon vectors are not prone to incorporating mutations and can tolerate larger and more complex transgenes, including those containing repeat DNA motifs. Moreover, the use of *SB*-based gene delivery eliminates the risk of rearrangements of the expression cassette that, as part of a transposing unit of DNA, integrates into chromosomal DNA in an intact form [120]. Compared with retroviral systems, the *SB *vectors have an inherently low enhancer/promoter activity [117,118]. Inserting insulator sequences flanking the transcription units of the cargo to prevent accidental *trans*-activation of promoters of neighboring genes further increased the safety features of the *SB *system (Figure 3D) [117]. Notably, the transposase can be provided as messenger RNA, thereby reducing the risk of 'rehopping' of the transposon-based vector [96]. Chromosomal integration of *SB *transposons is precise and random (see above), and no *SB*-associated adverse effects have been reported [116,120,121]. Of note, a precise integration mechanism, random integration pattern and negligible promoter/enhancer activity do not appear to be general features of all recombinase/transposon systems. For example, integration promoted by the bacteriophage-derived *PhiC31 *system was reported to generate chromosomal rearrangements [122,123]. The 5' TIR of the *piggyBac *transposon exhibits significant promoter activity in mammalian cells [124], and its genomic integration profile resembles that of integrating viral vectors [12], as described above.

The past few years have seen a steady growth in interest in applying the *SB *system for the treatment of several conditions including haemophilia A and B [103,104,106,107,125], junctional epidermolysis bullosa [126], tyrosinemia I [127], Huntington disease [128] sickle cell disease [129], mucopolysaccharidosis [105,130], cancer [109,131] and type 1 diabetes [132]. In addition, important steps have been made towards *SB*-mediated gene transfer in the lung for potential therapy of α-1-antitrypsin deficiency, cystic fibrosis and a variety of cardiovascular diseases [108,133]. Thus, the establishment of non-viral, integrating vectors has generated considerable interest in developing efficient and safe vectors for human gene therapy [116,120,134-136].

The *SB100X *hyperactive transposon system yields efficient stable gene transfer after non-viral gene delivery into therapeutically relevant primary cell types, including stem or progenitor cells. For example, the use of the *SB100X *system yielded robust gene transfer efficiencies into human hematopoietic progenitors [27,110], mesenchymal stem cells, muscle stem/progenitor cells (myoblasts) and iPSCs [137]. These cells are relevant targets for stem cell biology and for regenerative medicine and gene- and cell-based therapies of complex genetic diseases. Importantly, expression of the *SB100X *hyperactive transposase did not adversely influence the differentiation or function of these adult stem/progenitor cells, nor was there any evidence of any cytogenetic abnormalities [137]. In the context of iPSC technology, the ability to coax the differentiation of pluripotent stem cells into clinically relevant, transplantable cell types is a key step towards their ultimate use in clinical applications, especially because undifferentiated iPSCs pose an intrinsic tumorigenic risk [138]. It was recently demonstrated that *SB *transposon-mediated delivery of the myogenic PAX3 transcription factor into iPSCs coaxed their differentiation into MyoD+ myogenic progenitors and multinucleated myofibers [137], suggesting that PAX3 may serve as a myogenic 'molecular switch' in iPSCs, a finding that has implications for cell therapy of congenital degenerative muscle diseases, including Duchenne muscular dystrophy.

The first clinical application of the *SB *system is currently ongoing using autologous T cells genetically modified to redirect specificity for B-lineage malignancies [139]. Lymphocytes are a suitable initial platform for testing new gene transfer systems, as there have been hundreds of infusions of clinical-grade T cells genetically modified using viral and non-viral approaches without apparent genotoxicity [140]. The *SB *transposon tested in the first human application carries a chimeric antigen receptor (CAR) to render the T cells specifically cytotoxic toward CD19-positive lymphoid tumors [141,142]. The advantage of using the *SB *system for the genetic modification of T cells includes the reduced cost associated with manufacturing of clinical-grade DNA plasmids compared with recombinant viral vectors. This is particularly important when one considers that trials infusing CAR-positive T cells are only now beginning to demonstrate anti-tumor effects [143,144]. The higher enzymatic activity of *SB100X *might enable integration efficiencies comparable with that of retroviral vectors to be achieved for next-generation trials.

## Conclusions

Transposon-based technologies have enormous potential to develop powerful genomic tools with the vision of creating a bridge between physiology and genetics and to establish safe and inexpensive protocols for clinical gene transfer. Simple, plasmid-based vectors matched by a corresponding transposase source offer an easy and efficient method for germline transgenesis in laboratory animals and in large animal species for biotechnology. Furthermore, it is now both accessible and practical to generate highly complex libraries of gene knockouts in model species with a view to establishing new models of human disease for the annotation of disease pathways and for therapeutic and pharmaceutical intervention. The recently developed *SB100X *hyperactive transposon system yields highly efficient stable gene transfer after non-viral gene delivery into therapeutically relevant primary cell types, including stem cells, and thus may facilitate the clinical implementation of *ex vivo *and *in vivo *gene therapies. The next phase of preclinical research will focus on further refinement in large animal models to undertake *SB*-mediated transposition *in vivo *and to improve the safety profile of *SB *vectors by target-selected transgene integration into genomic 'safe harbors'. Although it remains to be seen whether the first clinical application of the *SB *system will result in a therapeutic effect, this trial will help validate the safety of this approach. The ongoing investigations will certainly prompt new ideas and new designs to be developed in this (ever) expanding universe of transposon technologies for genetic and cell engineering.

## Competing interests

The authors declare that they have no competing interests.

## Authors' contributions

Both authors contributed to drafting, reading and approving the final manuscript.
